# Analysis of the Anatomic Relationship Between the Mandibular First Molar Roots and Mandibular Canal Using Cone-Beam Computed-Tomography in 101 Dogs

**DOI:** 10.3389/fvets.2019.00485

**Published:** 2020-01-17

**Authors:** Jamie A. Berning, Christopher J. Snyder, Scott Hetzel, David P. Sarment

**Affiliations:** ^1^Midwest Mobile Veterinary Dentistry, Dublin, OH, United States; ^2^Veterinary Dentistry and Oral Surgery, Department of Surgical Sciences, University of Wisconsin-Madison School of Veterinary Medicine, Madison, WI, United States; ^3^Department of Biostatistics and Medical Informatics, University of Wisconsin-Madison, Madison, WI, United States; ^4^Xoran Technologies, LLC, Ann Arbor, MI, United States

**Keywords:** canine, mandible, mandibular molar, tooth root, computed tomography

## Abstract

The mandibular first molar (M1) tooth of the dog is commonly involved in dental procedures. Tooth roots and the mandibular canal can vary in location, which has not been described on a large scale. The objective of this study was to describe the three-dimensional anatomic relationship of the mandibular M1 tooth roots and the mandibular canal in dogs. Cone-beam computed tomography (CBCT) was used to evaluate the anatomic relationship between the M1 tooth roots and the mandibular canal. CBCT images were collected from 101 canine cadaver heads from a variety of unknown breeds. All skulls used in this study were mesaticephalic, confirmed by facial index calculations. The position of the apex in relation to the mandibular canal and in relation to the buccal and lingual cortices was recorded and analyzed in relation to mandibular bone height: root length ratio. When evaluating the apex in a buccal-lingual relationship, the tooth roots were found to be located closer to the lingual cortex in 73.3% of M1 roots. Tooth root apical positions were found to be symmetric between the right and left side of the mouth in 93% of mesial roots and 95% of distal roots. Apical positions relative to the mandibular canal within the same tooth were found to be consistent in 52% of teeth. Teeth with roots dorsal to the mandibular canal were associated with the largest mandibular bone height: root length ratio. CBCT provides a more precise overview than dental radiographs of three-dimensional anatomy. The tooth root position can be estimated in a clinical setting based on the ratio of mandibular bone height to tooth root length obtained from intraoral radiographs. Understanding the relative location of important anatomic structures is key to avoiding complications associated with various dental procedures. This study has documented that assessing anatomic structures with 2D imaging alone is flawed, and the large majority of dogs have M1 roots closer to the lingual aspect than the buccal aspect of the mandible.

## Introduction

The carnassial teeth are recognized in dogs as the maxillary fourth premolar (PM4) and mandibular first molar (M1) teeth and are strategic teeth with an important role in chewing and grinding food (1). The M1 is a large two-rooted tooth. The mesial two-thirds of the crown is similarly shaped to the premolars and is intended for shearing whereas the distal one-third of the crown is flat for grinding (2). The surface area of the mesial root has been described to be larger than the distal root (3).

The apices of the roots of the M1 tooth are reported to vary in location in relation to the mandibular canal (4, 5). M1 is commonly affected by severe periodontal disease which can necessitate exodontia or can lead to pathologic jaw fractures (6–8). The close relationship between the M1 tooth roots and the mandibular canal is an important consideration when surgically manipulating structures in this location since the mandibular canal contains the inferior alveolar neurovascular bundle. Disruption of these structures can result in hemorrhage, paresthesia, pulp necrosis, and tooth nonvitality rostral to the disruption (9–11).

Understanding the anatomic relationship between M1 tooth roots within the mandibular canal is essential to extraction of this tooth, the safe retrieval of M1 fractured root tips, and surgical endodontic therapy (12). Until recently, many studies evaluating the tooth root anatomy were limited to two-dimensional imaging. However, this approach has limitations, particularly in the buccolingual direction, since projections and angulations result in difficult interpretation of the anatomy due to superimposition (13–16). Consequently, a better understanding of the apex-to-mandibular-canal relationship will minimize risk for iatrogenic trauma to the canal contents. The use of three-dimensional imaging allows for accurate assessment of tooth root shape and their association with nearby anatomic structures. Cone-beam computed tomography (CBCT) has been shown to be an important tool for evaluation of three-dimensional relationships of dentoalveolar and maxillofacial structures (7, 17–20). In human dentistry, CBCT has become more common in recent years due to ease of use and high spatial resolution (21). The purpose of this study was to precisely describe this anatomic three-dimensional relationship using CBCT in a large number of dogs, which may provide guidance for various types of treatment planning. We hypothesize that the position of the M1 tooth roots in both a buccal-lingual orientation as well as the apical position relative to the mandibular canal can both be predicted.

## Materials and Methods

One hundred and one canine cadaver heads were obtained from a commercial supplier of osteologic specimens^1^ for use in this study. The heads were derived from a random variety of unknown dog breeds, ranging in approximate sizes from toy to giant breeds. Ethical approval for this study was not required according to national legislation because specimens acquired were humanely euthanized and commercially available for purposes unrelated to this study.

Cone beam computed tomography (CBCT) scanning was performed using a veterinary mobile unit^2^. Each head was placed onto a mock-surgical table using the carbon-fiber radio-clear extension platform designed for the scanner. The head was rested in a foam cradle and foam wedges were used, as needed, for proper consistent positioning in left lateral recumbency. Positioning lasers within the scanner were turned on for best approximation of the specimen location, allowing the head to be positioned centrally in both vertical and horizontal planes within the gantry. A scout view was taken to ensure appropriate positioning for scanning, and any position adjustments were made to insure inclusion of all desired anatomical structures in the field of view. A standard-resolution scanning protocol was selected, resulting in isotropic 0.3 mm voxel sizes. Twenty second scans were immediately available on the device to ensure appropriate outcome. All further viewing and measurements were performed using the same acquisition software^3^. The examiner was free to adjust windows, levels, and zoom to assess structures. Tooth roots were excluded from the study if alveolar bone loss, indicative of periodontitis, or if bony proliferation were present. Normal tooth and bone anatomy, defined as alveolar crestal bone height at the level of the cementoenamel junction and smooth ventral cortex without evidence of proliferative bone, were considered necessary criterion in order to collect accurate measurements. Facial index was calculated by measuring the width of the skull between the zygomatic arches, the length of the skull between the nasion to the prosthion. The width was multiplied by 100 and divided by the length to obtain the index. Skulls were considered mesaticephalic if the facial index was within a range of 96–163 (22).

A series of measurements were taken from each specimen associated with the right (409) and left (309) M1 teeth and supporting mandibular bone. The transverse view was used for taking measurements for each scan with the mandibular tooth apices pointing downward. Measurements were taken on the image slice in which the root appeared the longest.

The positional relationship of the tooth root and mandibular canal were categorized by a single observer (JAB) as follows:

B: the apex was either dorsal or adjacent to the mandibular canal and closer to the buccal cortical surface than the lingual cortical bone surface.
L: the apex was either dorsal or adjacent to the mandibular canal and closer to the lingual cortical surface than the buccal cortical bone surface.
A: the apex was dorsal to the mandibular canal and equidistant from buccal and lingual bone plates.
M: the apex was located inside the mandibular canal.

Due to the very few occurrences of some of the possible positions, analysis was simplified to lingual = “L” or “other” (B, M, or A).

**Figure 1:**
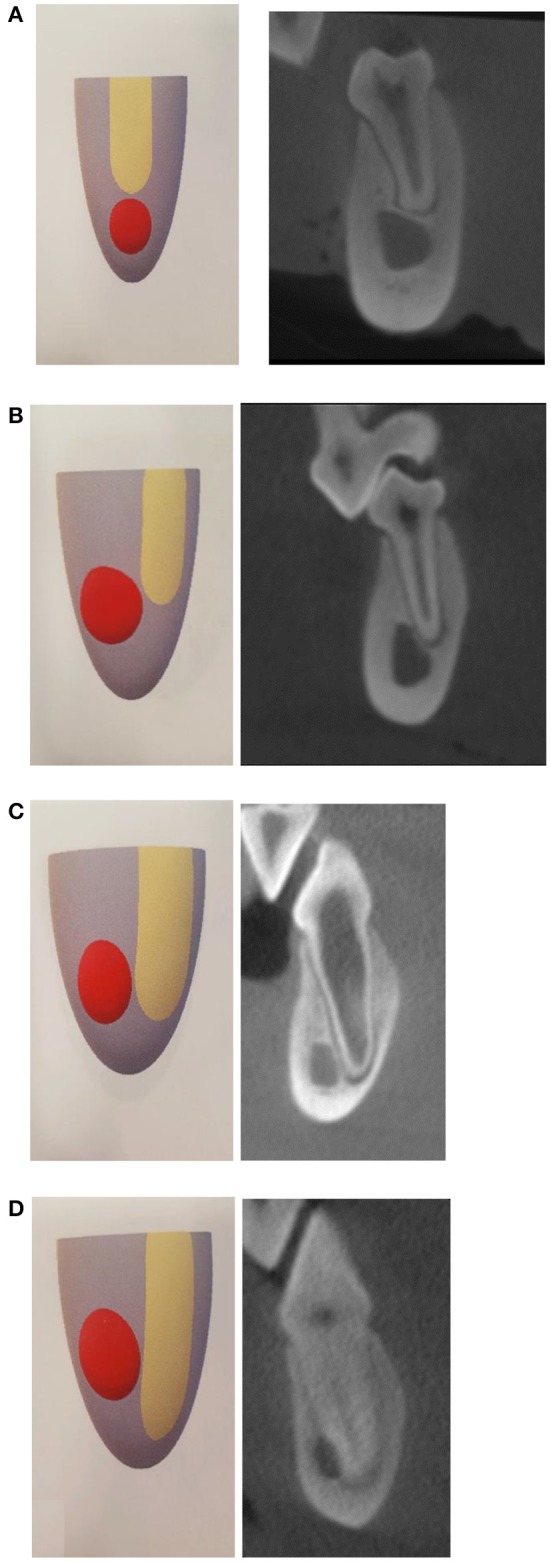
Apical positioning relative to the mandibular canal is classified as four different locations. **(A)** Apical position 0 is described as the apex terminating dorsal to the mandibular canal. **(B)** Apical position 1 is described as apices extending or contacting up to 50% the height of the mandibular canal. **(C)** Apical position 2 includes apices extending >50% the height of the mandibular canal but not extending ventral to the mandibular canal. **(D)** Apical position 3 is described as apices that extend ventral to the mandibular canal.

The extent of apical overlap with the mandibular canal was measured and recorded and denoted as follows (Figures 1A–D):

Position 0: the apex is dorsal to the mandibular canal.
Position 1: the apex and root contacts up to one-half of the side of the mandibular canal height.
Position 2: the apex and root contacts more than one-half of the mandibular canal height, but does not extend ventrally past the mandibular canal.
Position 3: the apex extends ventrally past the location of the mandibular canal.

Mandibular bone height was measured and recorded from the alveolar crestal bone height to ventral cortex. Root length measurements were taken from the cementoenamel junction to the apex. Differences in apical positions for mandibular bone height:root length ratio were assessed with a mixed effects ANOVA model with dog as a random effect and controlling for side (left, right) and root (mesial, distal) as fixed covariates. A similar model was run for comparison of mandibular height by apical position.

The positional relationship of the M1 tooth roots and the mandibular canal were evaluated by McNemar's test of paired binary variables. Kappa values were used to assess intra-dog correlation of M1 tooth roots and the mandibular canal, while weighted Kappa values were used for the relationships involving apical position. All analyses were conducted using *R* for statistical computing^4^. A *p* < 0.05 was considered to be significant.

## Results

A total of 101 unique specimens met the inclusion criteria for both (left and right) M1 teeth and both (mesial and distal) roots. Quantifiable information of interest included: root/apex buccal-lingual position, apical position relative to the mandibular canal, mandibular height, root length, and mandibular height:root length ratio. Data was collected for each root. All skulls used in this study were mesaticephalic, confirmed using facial index (mean 127.5, range 100–167).

Mandibular height ranged from 10.20 to 33.37 mm and had mean (SD) of 22.25 mm (4.96 mm). Root length ranged from 6.77 to 21.35 mm and had mean (SD) of 13.25 mm (2.87). Ratio of mandibular height to root length ranged from 1.07 to 2.72 and had mean (SD) of 1.70 (0.30). The mean mesial root length was 18.74 mm (8.25–21.35 mm) and distal root length was 16.67 mm (6.63–15.74 mm).

Buccal-lingual positions of apices relative to the mandibular canal presented in the following frequency: 329 roots were in position L and 71 were in position A. Two roots only were in positions B and position M each. Of the 71 roots that were classified as “A,” only 2 were in a dog with a mandibular height <20 mm (16.5 mm) with the remaining 69 roots in dogs with mandibular height >20 mm (20.4–32.1 mm). The two buccal roots were in a small dog with a mandibular height of 11.4 mm. The two roots that entered into the mandibular canal were in a small dog with a mandibular bone height of 11.1 mm.

### M1 Buccal-Lingual Relationships

When evaluating the buccal-lingual relationship between distal and mesial roots within the same tooth, 73.3% of teeth demonstrated both mesial and distal roots in the lingual position relative to the mandibular canal. Both the distal and mesial roots were both in “other” positions in 10.4% of teeth. In 9.4% of the teeth evaluated, the distal root was lingual and the mesial root was “other.” The distal root was in the “other” position with the mesial root in a lingual position in 6.9% of teeth (Table 1).

**Table 1:** Summary of lingual position of root pairs within each tooth.

	**All mesial roots**		**Center mesial roots**		**Right mesial roots**	
**Distal root**	**Lingual position**	**Other**	**Lingual position**	**Other**	**Lingual position**	**Other**
Lingual position	148 (73.3%)	19 (9.4%)	74 (73.3%)	11 (10.9%)	74 (73.3%)	8 (7.9%)
Other	14 (6.9%)	21 (10.4%)	6 (5.9%)	10 (9.9%)	8 (7.9%)	11 (10.9%)
McNemar's p	0.486		0.332		1	
Kappa (95% CI 95%)	0.460 (0.323–0.598)		0.440 (0.247–0.632)		0.481 (0.286–0.676)	

*McNemar p-values indicate that discordant percentage is not statistically due to which root. This leads us to conclude that if one root is “lingual” or “other” then the other root will likely be a match. Kappa values indicate moderate agreement of lingual type between roots of the same tooth*.

The buccal-lingual relationship of roots between jaws (left and right M1) in the same dog was evaluated. When evaluating the right and left mesial roots within the same dog, 78.2% of the time both mesial roots of the same patient were in the lingual position. In 17.8% of teeth, the mesial roots of M1 teeth in both jaws of the same patient were in an “other” position. Mesial roots demonstrated consistent buccal-lingual location between the left and right sides of dogs 96.0% of the time. Only 4.0% of patients exhibited roots in different positions when comparing the right and left sides of the mouth (Table 2). When evaluating right and left distal roots within the same dog, 81.2% of the roots were both in a lingual position while 15.8% of the specimens had distal roots in a position other than lingual. Overall, 97.0% of dogs had right to left symmetry with the distal root in the same buccal-lingual position, while only 3.0% had non-matching positioning of the distal root between the right and left sides (Table 3). The kappa value indicates an excellent agreement of lingual or other position between left and right side of the mouth in the mesial and distal roots within the same dog.

**Table 2:** Evaluation of the same buccal-lingual relationship between the left and right mesial tooth roots for each dog.

**Mesial roots**	**Right mesial roots**	
**Left mesial roots**	**Lingual**	**Other**
Lingual	79 (78.22)	1 (0.99)
Other	3 (2.97)	18 (17.82)
McNemar's p		0.617
Kappa (95% CI)		0.875 (0.681–1.000)

*McNemar p-value indicate that discordant percentage is not statistically due to the side of the mouth. Kappa value indicates excellent agreement of lingual or other position between left and right side of the mouth in the mesial roots within the same dog*.

**Table 3:** Evaluation of the same buccal-lingual relationship between the left and right distal tooth roots for each dog.

**Distal roots**	**Right distal roots**	
**Left distal roots**	**Lingual**	**Other**
Lingual	82 (81.19)	3 (2.97)
Other	0 (0.00)	16 (15.84)
McNemar's p		0.248
Kappa (95% CI)		0.896 (0.703–1.000)

*McNemar p-value indicate that discordant percentage is not statistically due to the side of the mouth. Kappa values indicate excellent agreement of lingual or other position between left and right side of the mouth in the distal roots within the same dog*.

### M1 Apical Positions

Apical position frequency was as follows: 134 roots were in position 0, 184 were in position 1, 62 were position 2, and 24 were in position 3. Apical position between distal and mesial roots within the same tooth matched in consistency in 52% of dogs (Table 4A) and 42.1% of the teeth had less overlap with the mandibular canal of the distal root when compared with the mesial root. Evaluating each right and left M1 teeth separately, left teeth demonstrated consistent positioning between roots in the same tooth in 51.5% of dogs (Table 4B). Evaluating the right M1 teeth, 52.5% of roots demonstrated consistent orientation relative to the mandibular canal (Table 4C). Weighted Kappa values demonstrate strong agreement between mesial and distal root apical positions within the same tooth. When considering all teeth, 46.5% of teeth demonstrated consistent apical positioning either both in apical position 0 (above the mandibular canal) or position 1 (extending <50% the height of the mandibular canal).

**Table 4A:** The same apical position between distal and mesial roots within the same tooth.

	**All mesial roots**			
**Distal root**	**0**	**1**	**2**	**3**
0	41 (20.3%)	37 (18.3%)	4 (2.0%)	0 (0.0%)
1	11 (5.4%)	53 (26.2%)	28 (13.9%)	1 (0.5%)
2	0 (0.0%)	1 (0.5%)	7 (3.5%)	15 (7.4%)
3	0 (0.0%)	0 (0.0%)	0 (0.0%)	4 (2.0%)
Weighted Kappa (95% CI)			0.634 (0.512–0.755)	

**Table 4B:** Apical position of root pairs within each tooth—left teeth.

	**Left mesial roots**			
**Distal root**	**0**	**1**	**2**	**3**
0	21 (20.8%)	19 (18.8%)	2 (2.0%)	0 (0.0%)
1	5 (5.0%)	26 (25.7%)	14 (13.9%)	1 (1.0%)
2	0 (0.0%)	1 (1.0%)	3 (3.0%)	7 (6.9%)
3	0 (0.0%)	0 (0.0%)	0 (0.0%)	2 (2.0%)
Weighted Kappa (95% CI)			0.621 (0.450–0.792)	

**Table 4C:** Apical position of root pairs within each tooth—right teeth.

	**All mesial roots**			
**Distal root**	**0**	**1**	**2**	**3**
0	20 (19.8%)	18 (17.8%)	2 (2.0%)	0 (0.0%)
1	6 (5.9%)	27 (26.7%)	14 (13.9%)	0 (0.0%)
2	0 (0.0%)	0 (0.0%)	4 (4.0%)	8 (7.9%)
3	0 (0.0%)	0 (0.0%)	0 (0.0%)	2 (2.0%)
Weighted Kappa (95% CI)			0.646 (0.474–0.819)	

*(A-C) Weighted Kappa values indicate strong agreement of apical position between distal and mesial roots within the same tooth. Shaded boxes demonstrate consistent apical root position between the mesial and distal roots on the left (B) and right (C) M1 teeth*.

Comparisons of apical position between the left and right mesial roots within each dog demonstrated 93.1% of mesial M1 roots were in consistent positions relative to the mandibular canal (Table 5A). The apical relationship of right and left distal roots had consistent positioning in 95.0% of dogs (Table 5B). In individual dogs, both right and left mesial roots terminated in either apical position 0 or 1 in 65.3% of cases. Distal roots both terminated in apical position 0 or 1 in 82.2% of cases.

**Table 5:** Agreement of apical position of left and right roots for each dog.

**(A) Agreement of apical position between left and right mesial roots for each dog**.				
	**Right mesial roots**			
**Left mesial root**	**0**	**1**	**2**	**3**
0	24 (23.8%)	2 (2.0%)	0 (0.0%)	0 (0.0%)
1	2 (2.0%)	42 (41.6%)	2 (2.0%)	0 (0.0%)
2	0 (0.0%)	1 (1.0%)	18 (17.8%)	0 (0.0%)
3	0 (0.0%)	0 (0.0%)	0 (0.0%)	10 (9.9%)
Weighted Kappa (95% CI)			0.958 (0.763–1.000)	
**(B) Agreement of apical position between left and right distal roots for each dog**.				
	**Right distal roots**			
**Left distal root**	**0**	**1**	**2**	**3**
0	39 (38.6%)	3 (3.0%)	0 (0.0%)	0 (0.0%)
1	1 (1.0%)	44 (43.6%)	1 (1.0%)	0 (0.0%)
2	0 (0.0%)	0 (0.0%)	11 (10.9%)	0 (0.0%)
3	0 (0.0%)	0 (0.0%)	0 (0.0%)	2 (2.0%)
Weighted Kappa (95% CI)			0.954 (0.759–1.000)	

*(A,B) Weighted Kappa value indicates excellent agreement of apical position between left and right side of the mouth in the mesial (A) and distal (B) roots within the same dog*.

### Apical Relationship to Mandibular Bone Height and Bone Height: Root Length Ratio

Average mandibular bone height was 22.2 mm and ranged from 10.2 to 33.4 mm. The average root length was 13.5 mm and ranged from 6.8 to 21.4 mm. Based on these measures, the calculated mean bone height to root length ratio was 1.70, which ranged from 1.07 to 2.72.

**Figure 2:**
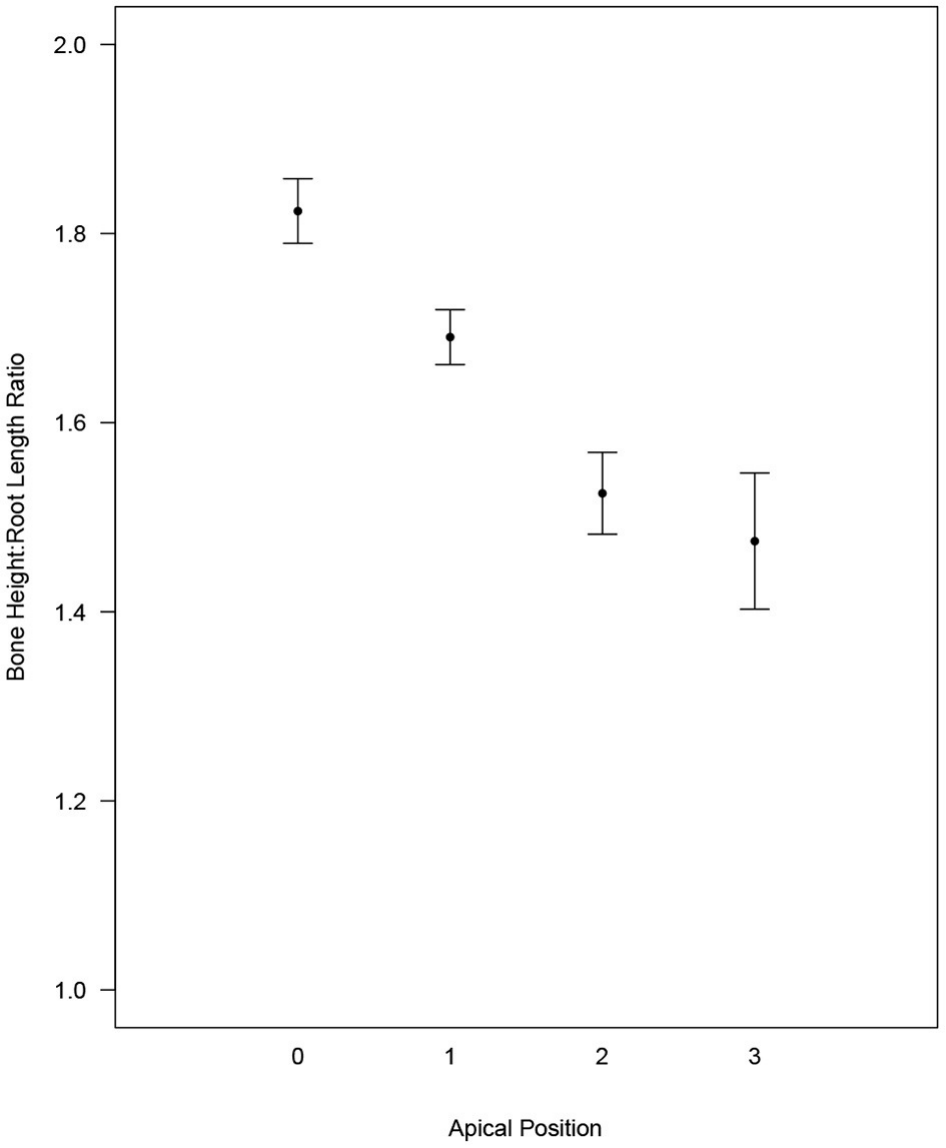
Estimated average ratio with CI for each apical position while controlling for multiple measures within each dog and for Side and Root. All two-way comparisons are statistically significant (Tukey *p* < 0.001) except for 2 vs. 3 (Tukey *p* = 0.476).

Based on the statistical model, which controlled for side (left or right) and root type (distal or mesial), the average (95% CI) ratio decreased when looking at apical positions 0 to 3 (position 0: 1.82 [1.79, 1.86]; position 1: 1.69 [1.66, 1.72]; position 2: 1.53 [1.48, 1.57]; and position 3: 1.47 [1.40, 1.55]). The ratio of the bone height:root length was statistically different between all 2-way apical position comparisons (Tukey *p* < 0.001), with the exception of position 2 compared to 3 (Tukey *p* = 0.476) (Figure 2).

**Figure 3:**
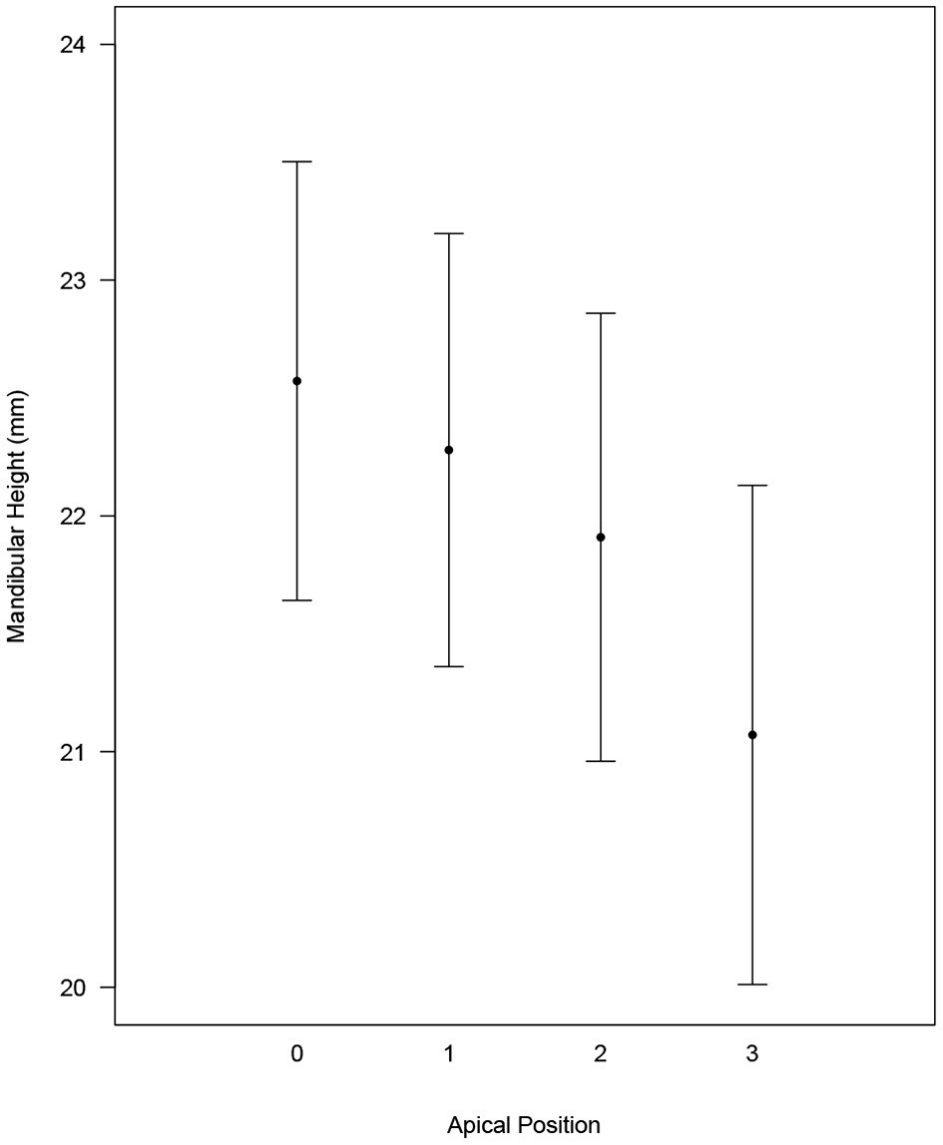
Estimated average mandibular height with CI for each apical position while controlling for multiple measures within each dog and for Side and Root. All two-way comparisons are statistically significant (Tukey *p* < 0.01) except for 0 vs. 1 (Tukey *p* = 0.103) and 1 vs. 2 (Tukey *p* = 0.114).

**Figure 4:**
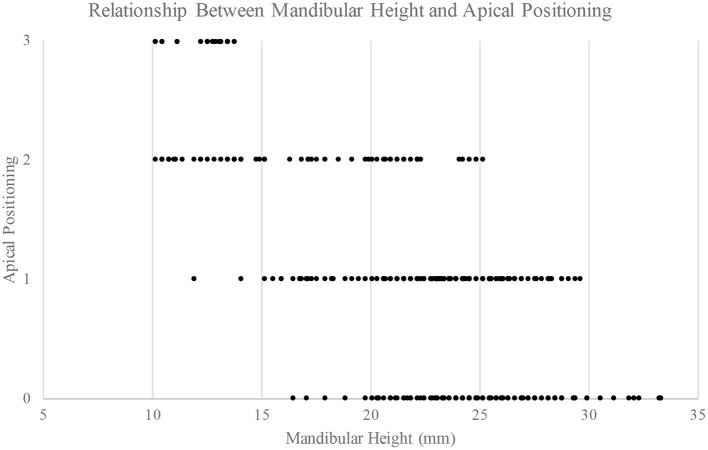
Relationship between mandibular bone height (mm) and apical position of tooth roots.

Based on a similar statistical model as used for the ratio, the average mandibular height also decreased from position 0 to 3 (position 0: 22.6 [21.6, 23.5]; position 1: 22.3 [21.3, 23.2]; position 2: 21.9 [20.9, 22.9]; and position 3: 21.1 [20.0, 22.1]). Mandibular bone height relative to apical position was found to be different (Tukey *p* < 0.01) when comparing all apical positions except position 0 vs. 1 (Tukey *p* = 0.103) and 1 vs. 2 (Tukey *p* = 0.114) (Figure 3). Mandibular bone height of 15 mm or less showed an apical position of 2 or 3 for 96.2% of roots. Mandibular bone height between 15 to 20 mm resulted in apical position 1 or 2 for 90.0% of roots. When mandibular bone height was >20 mm, 51.6% of roots were in position 1, 41.6% were in position 0, and 6.8% were in position 2 (Figure 4).

## Discussion

This study reports that 73.3% of M1 teeth evaluated had apices located closer to the lingual cortical bone surface than the buccal cortical bone surface. This finding is helpful when planning a surgical approach for a retained tooth root or surgical endodontic therapy to reduce or avoid complications associated with disruption of the mandibular canal and neurovascular bundle. A dog with a smaller mandibular height:root length ratio, and typically a smaller mandibular bone height, is more likely to have overlap of the tooth root with the mandibular canal. Therefore, the knowledge of tooth root position in relation to the mandibular canal becomes especially important in smaller dogs.

While 52% of dogs had mesial and distal roots at the same apical position, 42.1% of the teeth had less overlap with the mandibular canal of the distal root than the mesial root. This is consistent with the finding that the mean mesial root length (18.74 mm) is greater than the mean distal root length (16.67 mm), which is also consistent with the previous literature (3). A combination of the shorter mandibular bone height and shorter root length in the area of the distal root, combined with anatomic variation of the location of the mandibular canal within the mandible likely contribute to the higher rate of occurrence (82.2%) where the distal root apices are either found to be in apical position 0 or position 1 as compared to the frequency in the similar position for the mesial roots (65.3%). This may play a role of clinical importance considering the mesial root apices occur in apical positions 2 and 3 more frequently than the distal root which, coupled with the lingual positioning of the root, suggests intraoperatively encountering the mandibular canal is more likely with the mesial root. Dogs with smaller mandibles and lower mandibular height:root length ratios are very likely to have tooth roots located lingually in positions 2 and 3. Encountering the mandibular canal is likely when removing bone on the buccal aspect of one of these tooth roots. This should be considered when making treatment recommendations and advising clients of possible complications, especially in smaller patients.

A comparison of apical position between mesial and distal roots on the left and right M1's within the same dog had a 93.6 and 95.0% agreement, respectively. It is likely, then, that when the apical position of the root of one side is determined, it can be assumed bilateral symmetry exists with a high likelihood that the contralateral mesial or distal root will be in the same apical position. This may be clinically useful information when bilateral procedures are performed.

A statistically significant difference (*p* < 0.001) was found between apical positions when evaluating bone height:root length between all two-way comparisons except 2 vs. 3 (*p* = 0.476). It is unsurprising that the apical position of “0” which has no overlap with the mandibular canal is seen in teeth with a relatively shorter root lengths compared with the height of the mandible. The apical position of “3,” is most commonly seen when the length of the root is similar to the height of the mandible (typically in smaller dogs), with a ratio approaching 1.0. The authors believe there is clinical importance in the ability to evaluate the bone height:root length ratio and determine extent of overlap since many of the apices are located on the lingual aspect of the mandibular canal.

Since breed and weight of the dogs in the study were unknown, reliance on measurement of mandibular height creates a clinically applicable scenario to help predict apical root position when CT is not available. The taller the mandible, the more likely the apical root position is to have less overlap with the mandibular canal. Conversely, the shorter the mandibular height (and therefore, the smaller the dog) the more likely the dog is to have tooth roots that overlap the mandibular canal. While these estimations can be accurately measured with CT, intraoral radiography may demonstrate a similar ratio. This ratio may be less precise due to positioning distortion.

Most mesaticephalic dogs demonstrate consistent left to right positioning of the M1 roots. Nearly three-quarters of dogs have apices that are located more lingual than buccal. This information should be useful for treatment planning and execution. Care should be used in applying this knowledge to dogs with different skull conformations, and further study is needed to evaluate whether these findings can be extrapolated to other types of skulls. Further evaluating the effect of size of the patient on the position of the roots in the buccal-lingual aspect is also important to verify the findings of this study where all size dogs were evaluated.

## Limitations

Since breed and weight of the dogs in this study were unknown, the most clinically applicable parameter to use for classification of dogs in this study was mandibular height to tooth root ratio. The clinical measurements of mandibular bone height as well as bone height:root length ratio can be evaluated using dental radiographs. Specific comparison between accuracy of measurements between CT and radiography warrant further investigation as well as evaluation into whether apical position varies between different skull shapes.

## Conclusions

A large number of M1 roots in mesaticephalic dogs are oriented closer to the lingual cortical surface and when the apex and mandibular canal overlap, the roots most commonly lie lingual to the mandibular canal. This knowledge may help the clinician minimize trauma to the mandibular canal.

Anatomic landmarks such as tooth root apices and the mandibular canal are easily visible using CBCT. In view of the anatomic variation, this study suggests that accurate imaging is important for correct diagnosis and surgical approach. While CBCT is becoming more popular in veterinary dental referral practices, most general practice veterinarians do not have 3D imaging on-site. This study confirms the location of M1 roots is predictable, and that dental radiographs can give an estimation of the tooth root location in a reliable manner for the majority of mesaticephalic patients.

## Data Availability Statement

All datasets generated for this study are included in the article/supplementary material.

## Ethics Statement

Ethical approval for this study was not required according to national legislation because specimens acquired were humanely euthanized and commercially available for purposes unrelated to this study.

## Author Contributions

JB and DS: project conception and design. SH: statistical analysis. JB and CS: initial draft of the manuscript. All authors contributed to manuscript revision, read and approved the submitted version.

### Conflict of Interest

DS was employed by company Xoran Technologies, LLC. The remaining authors declare that the research was conducted in the absence of any commercial or financial relationships that could be construed as a potential conflict of interest.

## Footnotes

^1^Skulls Unlimited, Oklahoma City, OK.

^2^VetCAT™, Xoran Technologies, Ann Arbor, MI.

^3^VetCAT™, Xoran Technologies, Ann Arbor, MI.

^4^R. The R Project for Statistical Computing, version 3.5.3.
